# Time-restricted eating effects on performance, immune function, and body composition in elite cyclists: a randomized controlled trial

**DOI:** 10.1186/s12970-020-00396-z

**Published:** 2020-12-11

**Authors:** Tatiana Moro, Grant Tinsley, Giovanni Longo, Davide Grigoletto, Antonino Bianco, Cinzia Ferraris, Monica Guglielmetti, Alessandro Veneto, Anna Tagliabue, Giuseppe Marcolin, Antonio Paoli

**Affiliations:** 1 Department of Biomedical Science, University of Padova, Nutrition and Exercise Physiology Lab – Via Marzolo, 3, 35131 Padova, Italy; 2 Department of Kinesiology & Sport Management, Texas Tech University, Lubbock, TX USA; 3 Sport and Exercise Sciences Research Unit, University of Palermo, Palermo, Italy; 4 Human Nutrition and Eating Disorder Research Center, Department of Public Health, Experimental and Forensic Medicine, University of Pavia, Pavia, Italy

**Keywords:** Endurance, Elite cyclists, Inflammation, Immune system, Intermittent fasting

## Abstract

**Background:**

Although there is substantial interest in intermittent fasting as a dietary approach in active individuals, information regarding its effects in elite endurance athletes is currently unavailable. The present parallel randomized trial investigated the effects of a particular intermittent fasting approach, called time-restricted eating (TRE), during 4 weeks of high-level endurance training.

**Methods:**

Sixteen elite under-23 cyclists were randomly assigned either to a TRE group or a control group (ND). The TRE group consumed 100% of its estimated daily energy needs in an 8-h time window (from 10:00 a.m. to 6:00 p.m.) whilst energy intake in the ND group was distributed in 3 meals consumed between 7:00 a.m. and 9:00 p.m. Fat and fat-free mass were estimated by bioelectrical impedance analysis and VO_2max_ and basal metabolism by indirect gas analyzer. In addition, blood counts, anabolic hormones (i.e. free testosterone, IGF-1) and inflammatory markers (i.e. IL-6, TNF-α) were assessed.

**Results:**

TRE reduced body weight (− 2%; *p* = 0.04) and fat mass percentage (− 1.1%; *p* = 0.01) with no change in fat-free mass. Performance tests showed no significant differences between groups, however the peak power output/body weight ratio (PPO/BW) improved in TRE group due to weight loss (*p* = 0.02). Free testosterone and IGF-1 decreased significantly (*p* = 0.01 and *p* = 0.03 respectively) in TRE group. Leucocyte count decreased in ND group (*p* = 0.02) whilst the neutrophils-to-lymphocytes ratio (NLR) decreased significantly (*p* = 0.03) in TRE group.

**Conclusions:**

Our results suggest that a TRE program with an 8-h feeding window elicits weight loss, improves body composition and increases PPO/BW in elite cyclists. TRE could also be beneficial for reducing inflammation and may have a protective effect on some components of the immune system. Overall, TRE could be considered as a component of a periodized nutrition plan in endurance athletes.

**Trial registration:**

This trial was retrospectively registered at clinicaltrials.gov as NCT04320784 on 25 March 2020.

## Introduction

A proper nutrition strategy is an important aspect of elite sport physical training, as nutrient availability can not only influence energy expenditure, body composition, and performance, but also the immuno-response to exercise. In the last few years, the study of intermittent fasting (IF) has gained great popularity. IF is a dietary strategy that requires individuals to totally or partially reduce caloric intake for periods that range from defined times within a day to 1–3 whole days per week. Traditionally, the most common examples of IF have been religious fasting, particularly the pattern of food abstention during Ramadan [1]. However, in the last decade, IF has been used specifically to improve body composition and health status in both healthy and obese populations [2–5].

In sports, IF is not a popular strategy and is mainly linked to those athletes that follow the Ramadan restriction, in which the caloric consumption is allowed only during nighttime with a great influence on the circadian rhythm, hormonal regulation and also thermoregulation due to a limited fluid intake [6, 7]. However, a recent meta-analysis indicated that clear detriments were not observed in most physical performance parameters during Ramadan fasting [8]. In the same study, authors remarked that reduced physical performance during Ramadan may be caused by sleep disturbance and dehydration, and that these effects seem to be more pronounced in amateur than elite athletes. Despite this, it is well known that even small differences in performance are fundamental to determine the results of elite sporting events.

Moreover, due to the intensity of training sessions, elite athletes normally undergo cycles of physiological stress, which can perturb the immune system and promote inflammation [9, 10]. High intensity training, especially if conducted for longer than 90 min, temporarily impairs some immune variables (i.e. lymphocyte and neutrophils counts), leading to greater risk of illness and infection [11, 12]. In the last two decades, different strategies, including nutrition, have been tested to overcome the immunosuppressive response in athletes. Results from “immunonutrition” studies have, for example, highlighted the importance of carbohydrates and polyphenols to reduce the acute inflammatory response to exercise [13, 14]. In addition, nutrient availability can directly impact immune processes during exercise and recovery from intense exercise [15]. For instance, long-term reduction of energy intake has been associated with greater susceptibility to illness in Olympic athletes [16, 17]. Fasting has been consistently shown to reduce inflammation [18]; however, its effect on the immune system is still elusive. As reported in a recent meta-analysis [19], performing intense exercise during Ramadan results in a variation of the immunological response, but further studies are required to explore its potential regulatory role on the immune system.

Time-restricted eating (TRE) is a particular form of intermittent fasting, during which the normal period of fasting is 12–23 h per day. TRE can be performed with or without caloric restriction, and in the latter, individuals are allowed to consume their normal energy intake as long as they adhere to the specified window of time. We have recently demonstrated that 8 weeks of TRE does not impair muscular performance improvements in young untrained individuals performing resistance exercise training [20], can decrease fat mass and improve some health-related biomarkers (i.e. IL-6, TNF-α, Insulin, HDL-C, TG) in resistance-trained males [21], and does not impair lean mass gain or performance improvements in resistance-trained females [22].

To our knowledge, there is no evidence regarding the effect of TRE on endurance sports or the impact that this type of diet can have on the immune system. In endurance sports such as cycling, the power/weight ratio is a very important physical indicator for the athlete. The power expressed by an athlete reflects the speed of the race or the ability to climb a greater slope at the same speed. On the other side, lower body weight of the athlete can be an advantageous factor considering that a lighter cyclist spends less energy to maintain the same speed and therefore gets less tired; or, as climbing is the true essence of this sport, a lighter athlete should be faster during a climb. It therefore seems plausible that a dietary approach that allows weight loss, while maintaining the muscle functional characteristics, could be a winning strategy for endurance athletes. The aim of this study was to investigate the effects of 4 weeks of TRE, with a daily 16-h fast and 8-h feeding window, in a group of young elite cyclists. We hypothesized that TRE would reduce inflammatory markers and fat mass without affecting physical performance.

## Methods

Sixteen healthy young men (age: 19.3 ± 0.1 years; weight: 69.66 ± 6.11 kg; fat mass: 11.16 ± 1.99 kg) were recruited from 5 different elite cyclist teams from the Veneto region in Italy and randomly assigned to a time-restricted eating group (TRE; *n* = 8) or a standard diet group (ND; *n* = 8) using a computer-generated software. To be included in the study, subjects must have been cycling for at least three seasons in an elite team. Exclusion criteria were recent injuries, usage of steroids or corticosteroids or any other medical condition that could interfere with study procedures. Subjects characteristics for each group are described in Table 1. All the participants read and signed an informed consent form describing all the study procedures approved by the ethical committee of the Department of Biomedical Sciences, University of Padova, and conformed to standards of the current Declaration of Helsinki. Study analysis was conducted in a single blind design at the University of Padova.

**Table 1:** Subjects characteristics

	TRE (*N* = 8)	ND (*N* = 8)
Age (years)	19.38 ± 2.39	19.38 ± 1.60
Body weight (kg)	67.04 ± 5.03	72.27 ± 6.24
Height (cm)	175.0 ± 4.82	179.1 ± 5.83
BMI (kg/m^2^)	21.85 ± 1.65	22.47 ± 1.83
Fat mass (kg)	10.78 ± 1.40	11.53 ± 2.24
Fat free mass (kg)	56.26 ± 4.93	60.74 ± 5.72
Training Km on previous season	13,500 ± 1500	13,000 ± 1500

Data are presented as mean ± SD

### Experimental design

**Fig. 1:**
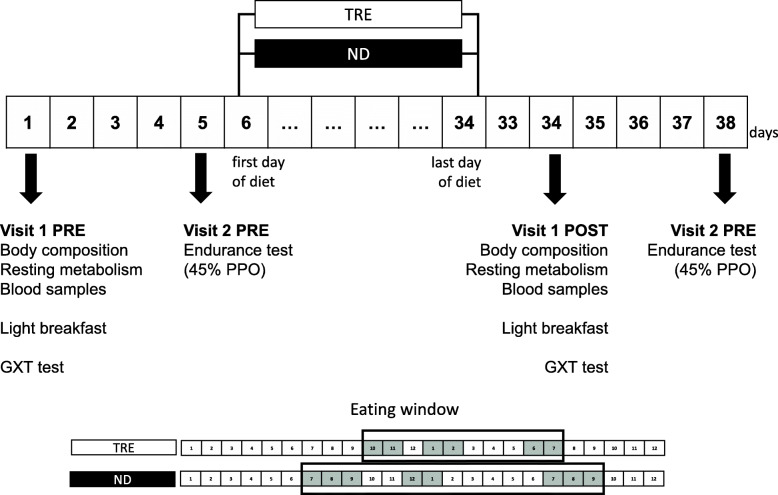
Experimental design. TRE, Time Restricted eating; ND, Normal Diet; GXT, Graded Exercise Testing, PPO, Peak Power Output

The experimental design is displayed in Fig. 1. All participants were tested on two different days before and at the end of the 4-week experiment. All participants started the experimental procedures in the month of January 2019.

Subjects reported to the laboratory after 12 h fasting, approximately between 7 and 9 am, and were asked to abstain from caffeine, alcohol consumption and from vigorous physical activity for 24 h prior to each measurement. Subjects were also asked to keep the same routine of dinner before each visits. At their arrival, body weight and height were measured and used to calculate body mass index (BMI). Fat mass (FM) and fat-free mass (FFM) were assessed by Bioelectrical Impedance Analysis (Human in Touch, DSMedica, Milano). Test–retest reliability for body composition obtained in our laboratory had an ICC of 0.99, consistent with previous findings [23]. Subjects were asked to lie down and rest for 10 min, whilst two electrodes were placed on their wrists and ankles. Through the analysis software, we obtained the values of FFM and FM.

Resting energy expenditure (REE) and oxygen uptake (VO_2_) were measured via a standard open-circuit calorimetry (max Encore 29 System, Vmax, Viasys Healthcare, Inc., Yorba Linda, CA, USA) on a breath-by-breath modality and estimated by the modified Weir equation as previously described [21, 24] . Participants rested in the supine position on a comfortable surface and stayed awake for the entire test duration. Following the application of a silicone mask that covered both mouth and nose, oxygen and carbon dioxide concentrations were obtained for a total of 20 min. Only the last 10 min were used to measure the results.

When the ventilatory test was completed, blood samples were collected from the antecubital vein, then aliquoted and stored at − 80 °C after proper centrifugation until analysis. All samples were analyzed by a certified laboratory using the same reagent lot. Complete blood count, total cholesterol, high-density lipoprotein cholesterol (HDL-C), low-density lipoprotein cholesterol (LDL-C), triglycerides (TG) were measured via enzymatic colorimetry (Modular D2400, Roche Diagnostics, Basel, Switzerland), insulin value was obtained with a chemiluminescent immunoassay (Siemens Immulite 2000), glucose was measured using the glucose oxidase method (glucose analyzer, Beckman Instruments, Palo Alto, CA, USA), interleukin-6 (IL-6), tumor necrosis factor-α (TNF-α) and interleukin-1β (IL-1β) were measured via Quantikine HS Immunoassay Kit (R&D Systems, Minneapolis, MN, USA), whilst insulin-like growth factor 1 (IGF-1) plasma concentrations were measured using the analyzer Liaison XL (DiaSorin S.p.A, Vercelli-Italy).

Thereafter, all subjects consumed a standard light breakfast of ~ 350–450 kcal mainly composed of carbohydrates. The exact same breakfast was provided during the post test. Approximately 45 min after breakfast, subjects performed an incremental maximal test (GXT) on a bike, in which peak power output (PPO; max watt maintained for at least 30 s), VO2 (l/min), VO2 max (ml/kg/min), carbon dioxide production (VCO2; l/min) and respiratory ratio (RR) were measured. Each athlete used personal shoes with Kéo pedals; the height of the saddle has been positioned at the maximum level of comfort for each athlete and for the subsequent tests the same relative measure was always used. Heart rate was recorded every 30 s, with a Polar band heart rate monitor (Kempele, Finland). After applying a silicone mask to the face for the analysis of respiratory gases, subjects started the test. The protocol included: 1 min of warm up at 60 W, then 1 min at 100 W, after which intensity increased by 30 W every 60 s until exhaustion.

Three to 4 days later, subjects performed an endurance test at 45% of the previously calculated PPO to measure the same ventilatory parameters. This test was chosen because it represents the exercise intensity normally registered during a long-distance race. Subjects were asked to cycle for 45 min at 45% of their PPO while respiratory gas analysis, VO2 (l / min), VO2 max (ml / kg / min), VCO2 (l / min), RR were obtained by the same gas analyzer used in visit 1. In addition, heart rate (HR) was collected at each minute of the test. Each athlete was asked to refrain from intense training on the day before the test and to consume a light and simple meal 2 h before the test. On average, subjects consumed approximately 350–450 kcal with a prevalent carbohydrate content (~ 50–80 g); specifically, a light breakfast of bread, jam, and fruit juice was provided. In order to reproduce the same conditions, all meals were recorded and repeated 28 days later during the POST test.

At the end of the second visit, all subjects received their diet protocol, and the study intervention officially started the following day. All laboratory testing procedures were repeated in the same order after 4 weeks of the intervention.

### Diet

During the recruitment phase, all subjects completed a validated 7-day food diary for athletes [25] which was analyzed by nutritional software (Dieta Ragionata 7.0) to estimate individual daily dietary intake. Based on the results from the food diary and specific energy and macronutrients requirements for athletes [26, 27], all athletes received the same 7-day diet plan, in which the caloric intake was set at 4800 kcal. The intervention dietary plan did not significantly differ from the athlete’s baseline regimen in terms of macronutrient intake, but the two intervention groups differed in the time window in which they consumed their meals (Table 2**)**. Before starting the trial, all participants reported consuming all meals between 7 and 9 am and 7–9 pm each day. The team dietitian supervised most of the meals consumed and ensured that all participants followed the prescribed diet intervention.

**Table 2:** Diet composition and macronutrients distribution

	TRE and ND	Breakfast		Lunch		Snack		Dinner	
		TRE	ND	TRE	ND	TRE	ND	TRE	ND
		10–11 a.m.	7–9 a.m.	1–2 p.m.	12–1 p.m.	With exercise		6–7 p.m.	7–9 p.m.
Total Energy intake (kcal/day)	4800.0	960.0		1680.0		480.0		1680.0	
Carbohydrates (g)	719.0	143.8		251.7		71.9		251.7	
Protein (g)	158.0	31.6		55.3		15.8		55.3	
Fat (g)	163.0	32.6		57.1		16.3		57.1	
Carbohydrates (%)	62.9								
Protein (%)	14.3								
Fat (%)	30.6								

Kcal for each macronutrient was calculated considering 3.5 kcal/g for protein, 3.75 kcal/g for carbohydrates and 9 kcal/g for fat

*TRE* time restricted eating, *ND* normal diet

The TRE group was instructed to consume 100% of their caloric intake divided in four eating occasions consumed in a time window of 8 h, with the three major meals distributed as follows: breakfast (10–11 a.m.), lunch (1–2 p.m.), dinner (6–7 p.m.). The ND group ingested their energy intake according to a traditional pattern, with breakfast between approximately 7–9 a.m., lunch at 12–1 p.m. and dinner between 7 and 9 p.m. Both groups consumed their snack either before or after the daily training session. Every week, a dietitian contacted subjects in order to check the adherence to the diet protocol.

### Training

The study was conducted during the winter pre-competition season. As such, most of the training consisted of long outings at a mild/medium pace at the steady state. The training schedule included 500 ± 50 km/week divided into 6 sessions per week that took place within the feeding time window (10 a.m.-6 p.m.).

### Statistical analysis

Statistical analyses were performed using the statistical software GraphPad Prism version 7.00 for Mac OS X (GraphPad Software). Target sample size was obtained assuming an interaction of a Root Mean Square Standardized Effect (RMSSE) of 0.25 with a fixed power of 80% and an alpha risk of 5% for the primary outcomes. Primary outcomes for the power analysis were VO_2_max, fat and fat-free mass. After assessing the normal distribution through the Shapiro–Wilk’s W test, an independent samples t-test was used to test baseline differences between groups. A two-way repeated-measures ordinary ANOVA was performed (using time as the within-subject factor and diet as the between-subject factor) in order to assess differences between groups over the course of the study. When the ANOVA model produced significant main or interaction effects, Post-hoc analyses were performed using the Bonferroni test. All differences were considered significant at *P* < 0.05.

## Results

### Body composition

**Fig. 2:**
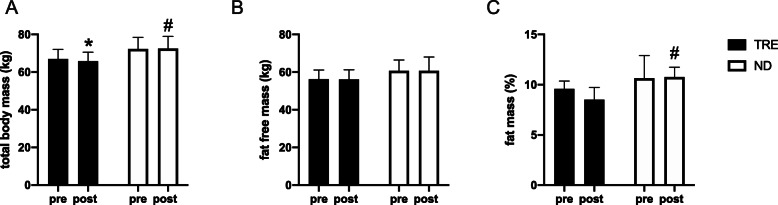
Body composition analysis. **a** total body mass; **b** fat-free mass and **c** fat mass. Data are mean ± SD * significantly different form pre values (*P* < 0.05); # significantly different form TRE value (*P* < 0.05). TRE, Time Restricted eating; ND, Normal Diet

After 4 weeks, a significant time x diet interaction (*p* = 0.04) was observed in total body weight: the TRE group experienced a significant (*p* = 0.03) decrease total body mass of ~ 2% (from 67.04 ± 5.03 to 65.78 ± 4.93 kg) while no significant change was observed in the ND group (from 72.28 ± 6.24 to 72.50 ± 6.45 kg). This alteration appeared to be mainly due to a decrease in total body fat mass. Although there was not a significant interaction, we observed a significant main effect of diet (*p* = 0.01) for the fat mass outcome. Relative fat mass decreased by ~ 11% (from 9.59 ± 0.77 to 8.52 ± 1.21%) in the TRF group but increased by ~ 4% (from 10.65 ± 2.24 to 10.77 ± 0.97% fat mass) in the ND group, although these changes were not statistically significant. Fat-free mass was maintained in both groups (TRE from 56.26 ± 4.93 to 56.15 ± 5.05 kg; ND from 60.74 ± 5.72 to 60.72 ± 7.37 kg) (Fig. 2).

### Resting metabolism

No statistically significant interaction was found for resting metabolism analysis. Resting energy expenditure (REE) calculated by Weir [24] equation decreased in both groups (main time effect *p* = 0.054; main diet effect *p* = 0.060) and to an apparently greater extent after the TRE diet (~ − 10%) compared to ND diet (~ − 2%). The analysis of ventilatory parameters showed a decrease of VO_2_ of about 10% in the TRE group (*p* = 0.07) and 4% in the ND group (*p* > 0.05). Similar results were obtained when VO_2_ was normalized by body weight (Table 3).

**Table 3:** Ventilatory measurement during resting, incremental and endurance test

	TRE		ND		2 Way ANOVA Time * diet	Main time effect	Main diet effect
	Pre	Post	Pre	Post			
**Resting metabolism**							
REE (Kcal/day)	1992 ± 251	1772 ± 147	2075 ± 273	2005 ± 109	ns	0.054	ns
VO_2_ (l/min)	0.28 ± 0.04	0.25 ± 0.02	0.30 ± 0.04	0.28 ± 0.02	ns	0.031	ns
VO_2_/kg (ml/kg/min)	4.18 ± 0.49	3.87 ± 0.46	4.05 ± 0.37	3.87 ± 0.43	ns	ns	ns
**Graded exercise testing (GXT)**							
VO_2_ (l/min)	4.83 ± 0.53	4.59 ± 0.56	4.90 ± 0.25	4.85 ± 0.26	ns	ns	ns
VO_2_/kg (ml/kg/min)	71.98 ± 3.93	69.94 ± 8.66	68.30 ± 7.19	67.17 ± 4.59	ns	ns	ns
VCO_2_ (l/min)	5.78 ± 0.49	5.44 ± 0.60	6.01 ± 0.39	5.71 ± 0.30	ns	0.028	ns
RR	1.19 ± 0.05	1.19 ± 0.05	1.23 ± 0.04	1.18 ± 0.03	ns	ns	ns
PPO (watt)	407.50 ± 41.66	415.00 ± 39.28	411.25 ± 35.63	403.75 ± 29.73	ns	ns	ns
PPO/BW (watt/kg)	6.08 ± 0.51	6.31 ± 0.46	5.71 ± 0.55	5.60 ± 0.53	0.024	ns	0.045
HR (bpm)	196.13 ± 9.99	190.63 ± 12.39*	190.88 ± 12.33	182.38 ± 10.07*	ns	0.0001	ns
**Endurance test (45% PPO)**							
VO_2_ (l/min)	2.95 ± 0.16	3.05 ± 0.38	3.00 ± 0.25	3.25 ± 0.56	ns	ns	ns
VO_2_/kg (ml/kg/min)	44.14 ± 2.68	46.13 ± 4.67	41.74 ± 4.53	45.11 ± 8.69	ns	ns	ns
VCO_2_ (l/min)	2.80 ± 0.19	2.90 ± 0.37	2.80 ± 0.31	2.94 ± 0.05	ns	ns	ns
RR	0.97 ± 0.04	0.96 ± 0.06	0.93 ± 0.05	0.91 ± 0.07	ns	ns	0.053
HR (bpm)	157.13 ± 16.86	149.00 ± 13.14*	158.13 ± 18.53	146.13 ± 14.46*	ns	0.0002	ns

Results are presented as mean ± SD

*TRE* time restricted eating, *ND* normal diet

* Significantly different from pre values (*p* < 0.05); # significantly different from TRE group (*p* < 0.05); ns (*p* > 0.05)

### Performance test

No significant differences were observed between groups in any of the performance test outcomes. Absolute PPO wasn’t affected by training or diet; however the PPO/BW ratio presented a significant time x diet interaction (*p* = 0.02), with a main effect of diet (*p* = 0.04) also observed; compared to the baseline value, the TRE group increased the ratio by 4% (*p* = 0.06) whilst ND decreased this ratio by 2% (*p* = 0.44), which leaded to a significant difference between groups (*p* = 0.02) in the post intervention values. Maximal heart rate achieved during the test equally decreased (*p* < 0.05 for time main effect) in both groups (TRE − 3%, *p* = 0.02; ND − 4%, *p* = 0.001). Similarly, during the endurance test performed at 45% of PPO, VO_2_, VCO_2_ and the RR remained unaltered whilst HR significantly decreased in both groups (TRE − 5%, *p* = 0.02; ND − 7%, *p* = 0.002) (Table 3).

### Blood analysis

Data from blood biochemistry analysis are presented in Table 4. Creatinine showed a significant time effect (*p* = 0.007) with a slight decrease of ~ 3% in the TRE group and a significant (*p* = 0.03) decrease of 5% after 4 weeks of ND. Ferritin was unaltered in the TRE group (+ 5%, *p* > 0.05) whilst apparently decreased in the ND group (− 23%, *p* = 0.053), although the time by diet interaction was not statistically significant.

**Table 4:** Blood biochemestry results

	TRE		ND		2 Way ANOVA Time*Diet	Main time effect	Main diet effect
	Pre	Post	Pre	Post			
**Complete blood count**							
RBC (10^6^/μL)	4.95 ± 0.38	4.90 ± 0.28	5.11 ± 0.40	5.09 ± 0.44	ns	ns	ns
HGB (g/L)	152.63 ± 11.50	148.13 ± 7.90	153.50 ± 10.53	151.38 ± 11.70	ns	ns	ns
HCT (%)	46.90 ± 2.80	44.41 ± 2.17*	47.01 ± 2.99	45.45 ± 3.24	ns	0.0035	ns
MCV (fL)	93.23 ± 1.98	90.60 ± 2.72*	92.08 ± 3.75	89.46 ± 4.17*	ns	0.0001	ns
MCH (pg)	30.85 ± 0.89	30.23 ± 1.05	30.06 ± 1.27	29.74 ± 0.98	ns	0.0069	ns
MCHC (g/L)	331.15 ± 9.66	334.04 ± 7.59	326.55 ± 6.84	332.89 ± 8.66	ns	ns	ns
RDW (%)	12.79 ± 0.66	12.91 ± 0.62	12.83 ± 0.31	12.65 ± 0.65	ns	ns	ns
Platelet (10^3^/μL)	231.38 ± 35.90	220.25 ± 45.86	244.25 ± 35.29	232.75 ± 43.75	ns	ns	ns
**White blood cells**							
Eosinophils (%)	3.06 ± 3.34	3.19 ± 3.06	3.63 ± 6.16	2.56 ± 2.09	ns	ns	ns
Basophiles (%)	0.75 ± 0.29	0.74 ± 0.24	0.44 ± 0.24	0.65 ± 0.40	ns	ns	ns
Monocytes (%)	6.83 ± 2.11	6.10 ± 1.47	6.25 ± 0.87	7.40 ± 1.21	0.0241	ns	ns
**Chemistry**							
Glucose (mg/dL)	94.63 ± 5.45	90.25 ± 6.54	91.00 ± 5.15	91.25 ± 6.94	ns	ns	ns
Creatinine (mg/dL)	0.86 ± 0.11	0.84 ± 0.10	0.85 ± 0.10	0.81 ± 0.10*	ns	0.0072	ns
Creatine kinase (mg/dL)	309.13 ± 227.94	273.38 ± 202.78	289.63 ± 388.26	231.50 ± 114.35	ns	ns	ns
Total cholesterol (mg/dL)	171.00 ± 18.52	181.38 ± 41.59	176.25 ± 17.56	179.38 ± 31.08	ns	ns	ns
TG (mg/dL)	69.75 ± 26.13	54.13 ± 20.23	70.50 ± 54.51	70.38 ± 56.89	ns	ns	ns
Iron (μg/dL)	110.13 ± 63.41	143.25 ± 30.16	97.63 ± 23.30	108.38 ± 37.39	ns	ns	ns
Ferritin (μg/L)	101.25 ± 28.34	101.75 ± 35.23	143.38 ± 57.67	110.63 ± 62.62	ns	ns	ns
Transferrin (mg/dL)	253.38 ± 23.20	243.13 ± 19.62	270.63 ± 29.42	257.63 ± 29.26	ns	ns	ns
CRP (mg/dL)	0.18 ± 0.17	0.08 ± 0.06	0.08 ± 0.06	0.07 ± 0.04	ns	ns	ns
ESR (mm/h)	2.75 ± 1.75	4.13 ± 2.10	2.00 ± 0.00	3.00 ± 1.07	ns	0.0138	ns
**Hormones**							
TSH (mcIU/mL)	2.23 ± 0.99	2.11 ± 1.34	1.79 ± 0.63	1.63 ± 0.46	ns	ns	ns
T3 free (pg/mL)	3.33 ± 0.42	3.13 ± 0.47	3.12 ± 0.40	3.39 ± 0.40	0.0114	ns	ns
Testosterone free (pg/mL)	33.73 ± 7.56	24.33 ± 7.23*	41.51 ± 14.09	38.33 ± 12.60#	ns	0.0045	0.0497
SHBG (nmol/L)	36.09 ± 13.85	32.73 ± 12.85	35.79 ± 10.69	31.98 ± 9.32	ns	0.0356	ns
Cortisol (ug/dL)	16.63 ± 2.79	11.78 ± 1.85*	15.48 ± 4.36	12.04 ± 4.74*	ns	0.0005	ns
Insulin (uIU/mL)	5.25 ± 1.75	3.88 ± 1.96	5.63 ± 1.60	5.50 ± 2.88	ns	ns	ns
IL-6 (pg/mL)	2.59 ± 1.13	2.24 ± 0.42	2.15 ± 0.42	2.73 ± 0.71	ns	ns	ns
Adiponectin (μg/mL)	3.00 ± 1.46	4.00 ± 2.24	4.63 ± 1.94	4.95 ± 2.30	ns	ns	ns
Adiponectin/FM (μg/mL/kg)	0.28 ± 0.14	0.39 ± 0.16*	0.43 ± 0.22	0.43 ± 0.22	ns	0.0488	ns
TNF (pg/mL)	7.26 ± 2.35	7.45 ± 2.99	7.24 ± 1.75	7.39 ± 2.84	ns	ns	ns
IGF-1 (ng/mL)	244.03 ± 90.83	291.60 ± 58.29*	329.60 ± 92.71	330.94 ± 62.71	ns	ns	ns

Results are presented as mean ± SD

*TRE* time restricted eating, *ND* normal diet

* Significantly different from pre values (*p* < 0.05); # significantly different from TRE group (*p* < 0.05)

The hormonal profile showed significant time x diet interaction (*p* = 0.01) in the levels of T3 free in which a slight decrease of ~ 6% was noted in the TRE whilst ND increased by ~ 9% (*p* = 0.07). Free testosterone decreased in both groups (main time effect *p* = 0.005; diet effect *p* = 0.05) but was significantly lower from baseline value only in the TRE group (− 27%, *p* = 0.006), such that at the post training time point, the two groups significantly differed (*p* = 0.03 TRE vs ND). We observed a trend in time x diet interaction (*p* = 0.07) in IL-6 value, with no change from baseline in the TRE group (− 3%) and a trend to increase after ND (+ 28%). On the contrary, adiponectin levels tended to increase (+ 33%, *p* = 0.08) after TRE diet whilst a lesser effect was noted in the ND group (+ 8%, *p* > 0.05). When adiponectin levels were normalized by body fat mass the differences showed an almost significant time x diet effect (*p* = 0.058), with TRE increasing by 50% (*p* = 0.02) and ND by 5% (*p* > 0.05). IGF-1 significantly decreased only in the TRE group (− 12%, *p* = 0.03) whilst no change was observed in the ND group (+ 3%, *p* > 0.05); as such we detected a trend to significance in time x diet interaction (*p* = 0.06).

**Fig. 3:**
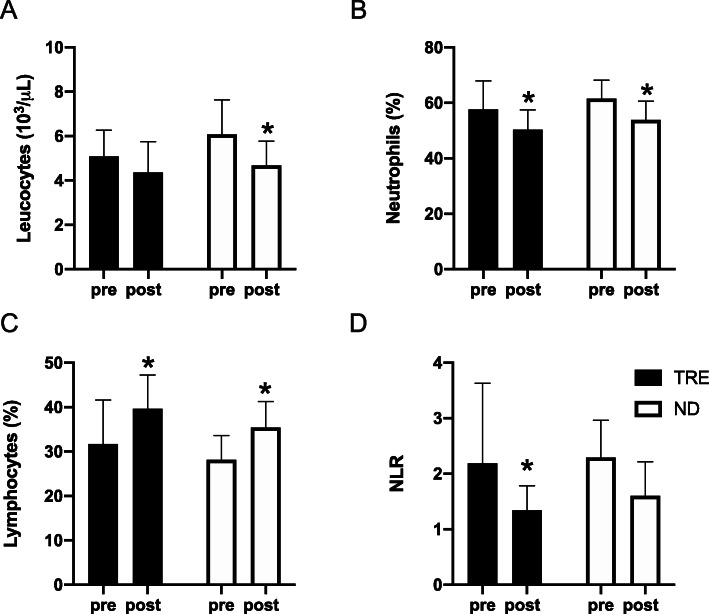
White blood cells response to 4 weeks of treatment. **a** White Blood Cells; **b** Neutrophils; **c** Lymphocytes and **d** Neutrophils-to-Lymphocytes ratio (NTR). Data are mean ± SD * significantly different form pre values (*P* < 0.05). TRE, Time Restricted eating; ND, Normal Diet

Leucocytes decreased in both groups (main time effect *p* = 0.001), but the difference between baseline and final values was only significant for ND (− 21%, *p* = 0.02 vs TRE − 14%, *p* > 0.05). The percentage of neutrophils significantly decreased by ~ 12% in both groups (*p* < 0.05), whilst lymphocytes increased by ~ 34% in the TRE group (*p* = 0.0004) and by ~ 27% in the ND group (*p* = 0.001). As a result, the Neutrophils-to-Lymphocytes ratio (NLR) decreased in both groups but was significantly different from baseline value only in the TRE group (*p* = 0.03) (Fig. 3).

## Discussion

TRE is a particular intermittent fasting approach in which the total daily caloric intake is often not reduced but distributed in a different frequency of meals, with the normal duration of eating ranging from 1 to 4 h to 10–12 h. In sports, intermittent fasting studies have mostly focused on Muslim athletes during the Ramadan period, a practice that requires abstention from food and liquid throughout the day and consumption of meals at night [6, 7]. To our knowledge, this is the first study that has investigated the effect of TRE in a group of elite endurance athletes. Our data demonstrated that during 4 weeks of pre-season training, TRE allows for maintenance of fat-free mass and reduction of fat mass, promoting a diminution of total body weight. We confirm that TRE reduced anabolic hormones (such as IGF-1 and testosterone) without affecting fat-free mass or endurance sport performance. In addition, it seems that TRE may exert a protective effect on some aspects of the immune system in the context of exercise training.

### Body composition

The TRE employed in this study was a 16/8 modality and consisted in 16 h fasting and 8 h feeding. We have recently demonstrated that the same TRE protocol used with resistance-trained athletes reduced total body weight and fat mass [21]. In the present study, we confirmed the positive effect of TRE on body composition. Total energy and nutrient intake were comparable between groups; for these reasons one possible explanation of the greater weight loss in the TRE group could be found in the increase in adiponectin. Adiponectin stimulates PGC-1α expression and mitochondrial biogenesis through the AMPK kinase [28], stimulating adipogenesis. Lower levels of adiponectin are associated with obesity [29] and oxidative stress [30]; whilst elevated plasma concentration of this cytokine correlates with improved metabolism and resting energy expenditure [28, 31]. We have previously demonstrated that adiponectin levels increase after 8 weeks of TRE compared to a normal diet; and these results correlated with weight loss [21]. In the present study, the levels of adiponectin tended to increase (+ 33%) after TRE compared to a blunted response in the ND group (+ 8%). When data were normalized relative to body fat mass, the apparent difference was even more pronounced (TRE + 50%; ND + 5%). Thus, TRE seems to induce a rise in adiponectin which may contribute to the reduction in body fat observed after 4 weeks of treatment. We have however observed a reduction in REE after TRE diet, which might seem a counterproductive adaptation. A decrease in REE is a common result of weight loss programs that involve calorie restriction, and it normally correlates with a loss of FFM and especially to changes in its composition [32, 33]. However, these studies are normally conducted in obese or overweight subjects not involved in any strenuous exercise program. In the present study, FFM was maintained and thus cannot explain the observed reduction in REE. It is, however, possible that this reduction reflects a momentaneous thermogenic adaptation of body weight, as described by the mechanical model of Rosenbaum and Leibel [34], in which REE decreases with initial weight loss. It is possible that in athletes, in which body fat is very low, and the exercise energy expenditure is particularly high, even a small drop of body weight (i.e 2–3%) may perturbate energy homeostasis. It is also possible that during TRE protocol, subjects have slightly reduced their caloric intake, which might have increased the negative energy balance and reduced REE. Each participant was strictly supervised by the personal team dietitian which ensured the adherence to diet protocol in terms of feeding window and caloric intake. Unfortunately, we have not recorded the post treatment dietary assessment to confirm or exclude this possibility.

Another explanation through which TRE may have improved body composition is linked to circadian clocks. The circadian control of food intake is situated in the hypothalamus which is synchronizes to the solar light–dark cycle. At the molecular level, the actions of specific genes (BMAL1, CLOCK) are deputed to control the cell-autonomous circadian rhythms. However, fasting and feeding alternation can directly impact daily circadian rhythms through the activation of mTOR, AMPK, CREB, and AKT, which are key regulators of metabolism and nutrient homeostasis [35]. A proper arrangement of nutrient intake and abstention can thus severely influence body metabolism. In a recent study, Yasumoto et al. [36] showed that a wrong pattern of feeding/fasting can desynchronize peripheral clocks inducing obesity and metabolic disorders in mice. It is plausible to assume that in our study, in which TRE subjects were forced to eat between 10 am and 7 p.m., the timing of feeding was well synchronized with the light–dark cycle and therefore may have positively impacted body metabolism. Unfortunately, we haven’t directly assessed the genes linked to the circadian clock, and further study should be employed to confirm this hypothesis.

### Aerobic performance and metabolism

In elite cyclists, the relation between PPO and body weight is an important contributing factor to sport performance. Overall, the performance during the incremental and endurance test did not change from baseline; however, we observed a significant increase of PPO/BW in the TRE group as compared to the ND group. It is interesting to note that fat-free mass wasn’t compromised during the diet regimen, even though TRE induced a reduction of testosterone and IGF-1. We have previously observed a drop of anabolic hormones after TRE [21], probably due to a reduced leptin-mediated control on the hypothalamo-hypophysial-gonadal axis. It seems indeed that intermittent fasting regimen can exert an inhibitory effect on the Leydig cells responsible for the production of testosterone [37, 38]. Moreover, it is possible that TRE, similarly to calorie restriction, stimulates the AMP-activated protein kinase/Acetyl-CoA-Carboxylase (AMPK/ACC) signaling pathway [3]. AMPK is a central metabolic regulator activated during low cellular energy status, when trigged it stimulates ATP production via fatty acid oxidation and glycolysis, while simultaneously inhibits anabolic processes. Studies performed in rodents, showed that short-term fasting (19–39 h) increases AMPK and ACC activation in adipocytes, but not in muscle [39, 40]; however, this hypothesis hasn’t been confirmed in humans. Moreover, caloric restriction doesn’t seem to reduce IGF-1 concentration, although it may increase IGFBP-1 [41]. In the present study we did not analyze plasma leptin but considering the similarity between the TRE protocol and our prior study in resistance-trained men, we can speculate that the reduction of anabolic hormones could be due to an alteration of adiponectin/leptin levels.

Unlike our previous study [21] on resistance-trained athletes, we didn’t observe any significant alteration of T3 nor TSH levels or REE in the TRE group. Interestingly, ferritin appeared to decrease in ND but not in TRE, although this was not statistically significant. Ferritin not only reflects iron stores but is also used as an index of exercise tolerance [42, 43]. A drop of ferritin in endurance athletes can occur during the initial months of training [44], although TRE seems to blunt this effect.

### Inflammatory markers and immune response

Endurance elite athletes present lower leucocytes counts, neutrophils and monocytes plasma concentration compared to other sports, probably due to the inflammatory adaptive response to aerobic training [45]. These decreases in components of the immune system may contribute to the susceptibility to bacterial and upper respiratory infection [46, 47]. Exercise increases pro-inflammatory cytokines (such as IL-6, IL-1β; TNF-α), which mediate the communication between immune and non-immune cells in order to elicit repair processes. This activation is transient, with markers normally returning to basal level in 24–30 h [48], and is followed by the release of anti-inflammatory cytokines that have the function to reduce the inflammatory process [49, 50]. As a result, the immune response temporarily decreases, with total leucocytes and neutrophils increasing whilst lymphocytes decrease. We already reported a modulation of some inflammatory markers after TRE (IL-6 and IL-1β and TNF-α) [21], whilst others did not observe any changes of these cytokines in healthy young men [51]. In the present study, IL-6 appeared to decrease in TRE and increase in ND (even if not significantly). Moreover, the ratio of neutrophils-to-lymphocytes (NLR) is a biomarker of systemic inflammation largely used in clinical practice, which correlates with the circulating level of C-reactive protein (CRP) [52, 53]. In our study, both groups presented a decrease in neutrophils and an increase in lymphocytes after 4 weeks of treatment, whilst the NLR seems to be more affected by the TRE compare to ND (TRE -39%; ND − 30%). Interestingly, while post-intervention leucocytes appeared lower than baseline in both groups, they were significantly reduced only in the ND group. It is known that short-term caloric restriction can compromise the immune system response [15, 54], however, the present experiment did not reduce the energy intake. This is the first time that TRE has been correlated to the immune response to exercise training, and it seems that TRE may possibly have a protective effect, attenuating the reduction in leucocytes count and thus potentially preventing the susceptibility to infections in young elite endurance athletes.

Some limitations of the present study should be taken into account. One is the reduced number of subjects involved, and secondly the usage of an interview methodology to determine energy and macronutrient composition. This approach has known weaknesses and may have played a role in the observed outcomes. Moreover, although the team dietitian supervised most of the meals consumed, we didn’t collect a food diary record during the intervention to compare with the baseline habits.

## Conclusion

Our results suggest that the 16/8 TRE protocol could be beneficial in elite endurance athletes to improve body composition and inflammatory markers without affecting aerobic performance. Moreover, TRE may help the function of the immune system, attenuating the decrease of leucocytes that typically occurs in high-trained individuals, although further investigation is warranted. This kind of dietary regimen could be adopted by endurance athletes to reduce body fat mass. Our preliminary data may suggest that TRE could be a proper dietary regimen in particular during the pre-season training that normally occurs during the winter season, in which the training-induced depression of immune system increases the respiratory infection susceptibility.

## Data Availability

The datasets during and/or analysed during the current study available from the corresponding author on reasonable request.

## Abbreviations

ACC: Acetyl-CoA-Carboxylase
AKT: Protein kinase B
AMPK: 5′ AMP-activated protein kinase
BMAL1: Brain and Muscle ARNT-Like 1
BMI: Body Mass Index
BW: Body weight
CLOCK: Basic helix-loop-helix-PAS transcription factor
CREB: cAMP response element-binding protein
CRP: C-reactive protein
FFM: Fat Free Mass
FM: Fat Mass
HDL-C: High-Density Lipoprotein Cholesterol
ICC: Inter class correlation
IF: Intermittent fasting
IGF-1: Insulin-like growth factor 1
IGFBP-1: Insulin-like growth factor Binding protein 1
IL-1β: Interleukin-1β
IL-6: Interleukin-6
LDL-C: low-density lipoprotein cholesterol
mTOR: Mammalian target of rapamycin
ND: Normal Diet
NRL: Neutrophils-to-Lymphocytes ratio
PGC-1α: Peroxisome proliferator-activated receptor gamma coactivator 1-alpha
PPO: Peak Power Output
REE: Resting energy expenditure
T3: Triiodothyronine
TG: Triglycerides
TNF-α: Tumor necrosis factor-α
TRE: Time Restricted Eating
TSH: Thyroid-stimulating hormone
VO2: Oxygen uptake
